# Dr. Frank R. Post

**Published:** 1913-02

**Authors:** 


Dr. Frank R. Post of Port Crane, committed suicide, Christmas Day, from despondency, his daughter having been burned to death a few months ago.

Our readers are requested to inform the families of deceased physicians that obituary notices will be inserted without charge. Portrait cuts will be used if desired or will be prepared from photographs at cost.

